# Retraction: SPARC Overexpression Inhibits Cell Proliferation in Neuroblastoma and Is Partly Mediated by Tumor Suppressor Protein PTEN and AKT

**DOI:** 10.1371/journal.pone.0228246

**Published:** 2020-01-29

**Authors:** The PLOS ONE Editors

After publication of this article [1] concerns were raised about the following figures:

- The GAPDH western blot panel for IMR-32 in Fig 1B appears similar to the GAPDH panel for IMR-32 in Fig 2B. These represent the same experimental conditions.
- In Fig 1E, all of the corresponding Non-IR panels appear similar for SK-N-AS and NB1691. In addition, the Non-IR Mock panels appear similar to the IR, SK-N-AS, pEV panel.
- The GAPDH panel for SK-N-AS in Fig 2B appears similar to the GAPDH panel for NB1691 in Fig 3A.
- The GAPDH panel for IMR-32 in Fig 4A appears similar to the GAPDH panel for NB1691 in Fig 6A.
- In the IMR-32 pc-Jun (ser 63) blot shown in Fig 5, when levels are adjusted the background in lane 3 appears different than that in other lanes.
- In Fig 7C, there appears to be a region of overlap between SPARC panels for Non-IR Mock (upper left quadrant) and Non-IR pEV (lower right quadrant).
- In S1B Fig., the GAPDH panels for SK-N-AS and NB1691 appear similar.

The University of Illinois at Chicago investigated this work and found evidence of data falsification involving Fig 1E.

In light of the above concerns that call into question the validity and reliability of the reported results and following the outcome of the institution’s investigation, the *PLOS ONE* Editors retract this article.

The authors either did not reply to the retraction notification or could not be reached.
